# The value of deep neural networks in the pathological classification of thyroid tumors

**DOI:** 10.1186/s13000-023-01380-2

**Published:** 2023-08-19

**Authors:** Chengwen Deng, Dan Li, Ming Feng, Dongyan Han, Qingqing Huang

**Affiliations:** 1 Department of Nuclear Medicine, Chaohu Hospital of Anhui Medical University, Heifei, 238000 China; 2 Department of Nuclear Medicine, Sun Yat‐sen Memorial Hospital, Sun Yat‐sen University, Guangzhou, 510289 China; 3 Tongji University, Shanghai, 200082 China; 4 Shanghai Tenth People’s Hospital Tongji University, Shanghai, 200072 China; 5 Shanghai Key Laboratory of Molecular Imaging, Shanghai University of Medicine and Health Sciences, Shanghai, 201318 China

**Keywords:** Deep neural network, Thyroid tumor, Pathology, Diagnostics, Artificial intelligence

## Abstract

**Background:**

To explore the distinguishing diagnostic value and clinical application potential of deep neural networks (DNN) for pathological images of thyroid tumors.

**Methods:**

A total of 799 pathological thyroid images of 559 patients with thyroid tumors were retrospectively analyzed. The pathological types included papillary thyroid carcinoma (PTC), medullary thyroid carcinoma (MTC), follicular thyroid carcinoma (FTC), adenomatous goiter, adenoma, and normal thyroid gland. The dataset was divided into a training set and a test set. Resnet50, Resnext50, EfficientNet, and Densenet121 were trained using the training set data and tested with the test set data to determine the diagnostic efficiency of different pathology types and to further analyze the causes of misdiagnosis.

**Results:**

The recall, precision, negative predictive value (NPV), accuracy, specificity, and F1 scores of the four models ranged from 33.33% to 100.00%. The area under curve (AUC) ranged from 0.822 to 0.994, and the Kappa coefficient ranged from 0.7508 to 0.7713. However, the performance of diagnosing FTC, adenoma, and adenomatous goiter was slightly inferior to other types of pathological tissues.

**Conclusion:**

The DNN model achieved satisfactory results in the task of classifying thyroid tumors by learning thyroid pathology images. These results indicate the potential of the DNN model for the efficient diagnosis of thyroid tumor histopathology.

**Supplementary Information:**

The online version contains supplementary material available at 10.1186/s13000-023-01380-2.

## Background

As the incidence of thyroid tumors is increasing year by year, it is extremely important to accurately diagnose their pathological types. The significant increase in the number of patients makes the doctor’s work burden heavier and work efficiency reduced. Common malignant thyroid tumors include PTC, MTC, and FTC, and benign nodules include adenomatous goiter and adenoma. All the above pathological tissues have varying degrees of similarity [1]. Once misdiagnosed, it will affect the subsequent treatment plan of patients [2]. Therefore, how to improve the efficiency of differential diagnosis of thyroid tumors has become a hot spot for current research.

The gold standard for thyroid tumor diagnosis remains pathology, but the method continues to face many challenges: (1) It takes years and months to train a good pathologist and cannot meet the rapid increase in surgical workload; (2) The varying levels of competence among pathologists and the uneven diagnostic accuracy; (3) The heavy workload can cause physician fatigue and increase the probability of misdiagnosis. Artificial Intelligence (AI) techniques has become the most eye-catching research hotspot in the field of science and technology, and AI software developed in large numbers in recent years has played an increasingly significant role in medical treatment. A large number of studies have now confirmed that AI can effectively address the above-mentioned problems. DNN models are good at learning intrinsic rules from large amounts of data. The application of high-efficiency DNN has become one of the important ways to solve the heavy clinical work. In particular, the rapid development of DNN models and their successful application in clinical settings have proven the ability to efficiently diagnose pathologies [3, 4] and improve the situation of misdiagnosis due to insufficient knowledge and fatigue of pathologists, playing an increasingly prominent role in medical care.

In summary, this study used DNN models represented by Resnet50, Resnext50, EfficientNet, and Densenet121 to diagnose different types of thyroid tumors, to analyze the causes of misdiagnosis of different pathological tissues, and finally to analyze whether DNN models have the potential to efficiently diagnose thyroid tumor pathology.

## Materials and methods

### Patients and data

The data for this paper were obtained from patients who underwent surgical treatment for thyroid nodules from July 2014 to August 2022. Inclusion criteria were: (1) Patients who underwent initial surgical treatment for thyroid nodules; and (2) Patients with clear pathology of thyroid nodules. Exclusion criteria were (1) Patients had no postoperative thyroid nodule pathology or with unclear pathology; (2) Patients had received ^131^I treatment; (3) Patients had received anti-tumor therapy. In total, there were 559 patients, including 381 PTC, 38 MTC, 41 FTC, 40 adenomatous goiter, and 59 adenoma. One or two pathology images were taken from each patients histopathology. 799 pathological images were collected, including 426 PTC, 40 MTC, 41 FTC, 44 adenomatous goiter, 59 adenoma, and 189 normal thyroid (189 cases were randomly selected from the above patients, and images of paracancerous tissue were collected as normal thyroid).

A total of 799 hematoxylin eosin (HE) stained pathological sections were used in this study. Specimens obtained by surgery or puncture were fixed in 4% neutral formaldehyde solution, dehydrated, paraffin-embedded, and stained with HE at a thickness of 4 μm in all patients. The Leica ASP300S fully enclosed tissue dehydrator was used for the dehydration process, and the Leica Auto Stainer XL automatic stainer was used for the staining process.

**Fig. 1:**
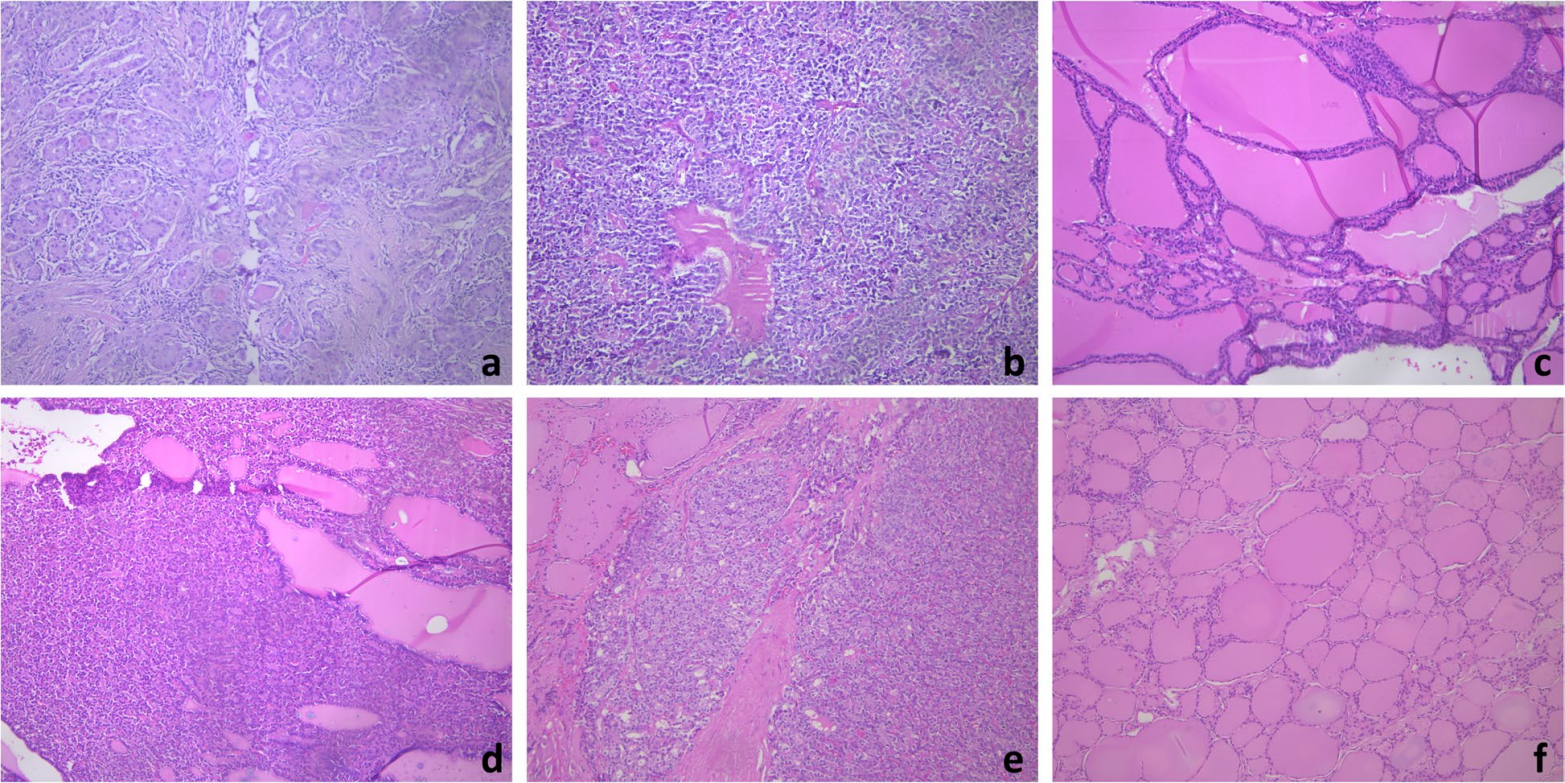
Pathological images of the thyroid gland. **a** PTC; **b** MTC; **c** Adenomatous goiter; **d** Adenoma; **e** FTC; **f** Normal thyroid

All pathological specimens were observed under a Leica DM4000B LED smart biomicroscope, and two highly qualified pathologists selected the area of interest and performed pathological diagnosis, selecting paracancerous tissue as normal thyroid tissue. The pathology images were captured manually and directly under the microscope using a Leica DFC495 microscope camera. Images that were controversial among physicians were excluded, and all pathology images were classified, as shown in Fig. 1. The acquired images were in TIF format and the average size of each image was 2500 pixels × 3200 pixels. The above instruments were manufactured by Leica Microsystems (Shanghai) Trading Co.

### Data augmentation

In order to achieve satisfactory classification results, data expansion of the original images is required, so data augmentation is performed on the pathology image data. We increase the amount of training data by random flip (50% probability of horizontal flip), random rotation (-10°-10°), random scaling (100%-110%), and random brightness enhancement (0–20%) on the images. For each image, only one of the four transformations is randomly applied.

### Network architecture

#### Densenet121

**Fig. 2:**
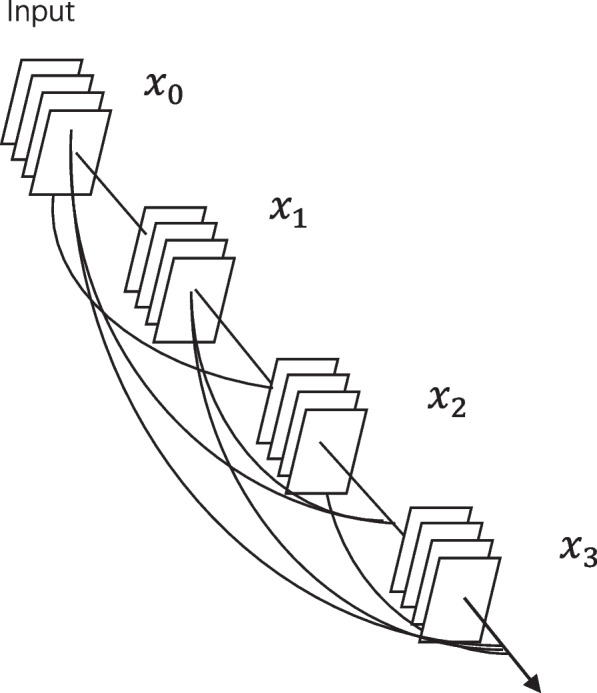
Structure diagram of Densenet121

In traditional CNN networks, the problem of gradient disappearance becomes more and more serious as the depth of the network deepens. The structure of the Densenet121 model mainly consists of multiple dense blocks, and each dense block is processed using ordinary convolutional layers between them. The dense block is composed of multiple convolutional blocks, each of which in turn consists of convolutional kernels, as in Fig. 2. And each dense block takes a skip connection between them, that is, the output of the previous dense block is directly passed to the output of the latter dense block, i.e., ${x}_{0}$, ${x}_{1}$, …, ${x}_{l-1}$. The output ${x}_{l}$ is obtained through the composite function ${H}_{l}$. This network structure effectively achieves dimensionality reduction and reduces the parameter computation with the following equation.

$$x_l=H_l(\lbrack x_0,\;x_1,\;...,\;x_{l-1}\rbrack)$$

#### Resnet50

**Fig. 3:**
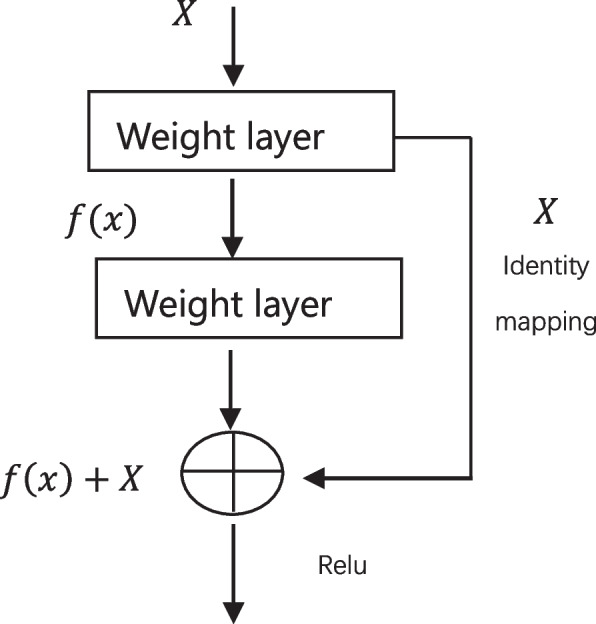
Structure of Resnet residual block

**Fig. 4:**
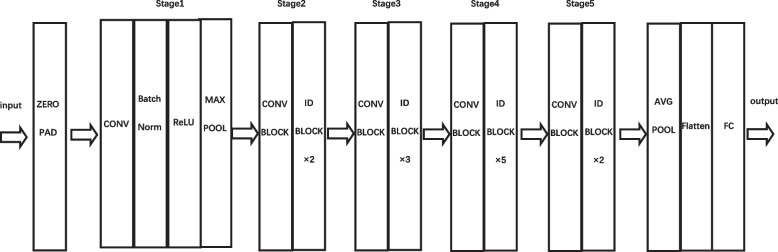
Structure of Resnet50

Resnet50 is obtained by modifying the VGG19 network, and the model incorporates a residual block through a shortcut mechanism (as in Fig. 3). The main function of the residual block is to create a shortcut between the input and the output, making it possible to train the network by learning only the upper part of the learning residuals instead of learning the whole process. This not only saves the transmission time of information from the input to the output, but also reduces the learning difficulty of the neural network. ResNet50 contains 49 convolutional layers and one fully connected layer, where ID BLOCK x2 in the second to the fifth stage represents two residual blocks without changing the size, and the structure is shown in Fig. 4. CONV is the convolution layer of the convolution operation, Batch Norm means the batch normalization process, Relu is the activation function, MAX POOL denotes the maximum pooling operation, AVG POOL indicates the global average pooling layer operation, and stage1 to stage5 represents the residual blocks. After continuous convolution operation of the residual blocks, the result is output by the Softmax classifier.

#### Resnext50

**Fig. 5:**
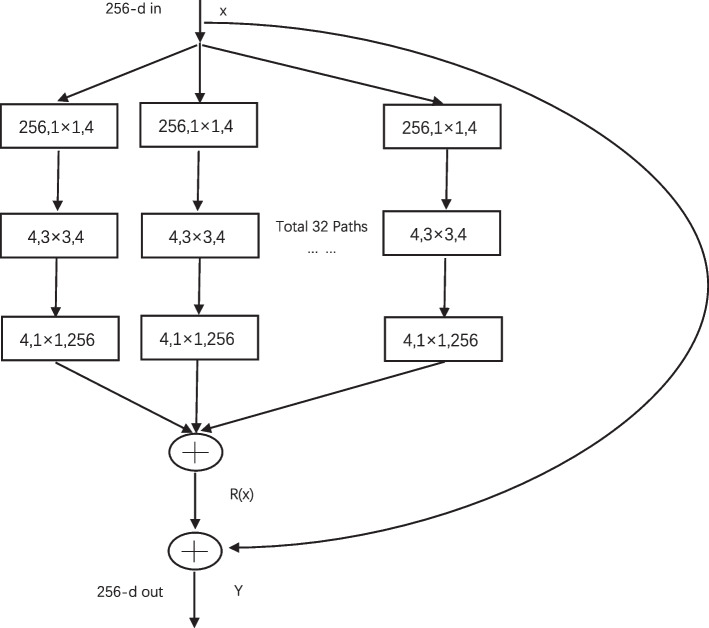
Structure diagram of Resnext50

ResNext is a special kind of residual network, which is a combination of a ResNet network and an Inception network. Its network block structure is composed of the simplified Inception structure block plus the shortcut of ResNet, which can guarantee the performance of the network while reducing the hyperparameters of the neural network. The structure is shown in Fig. 2. The essence of ResNeXt is group convolution, where the number of groups is controlled by the cardinality of variables, and the blocks of the original ResNet three-layer convolution are replaced by a parallel stack of blocks with the same topology. The network is designed to depart from the fixed mindset of improving network performance by deepening and widening the network hierarchy, and increases the number of paths with the same topology to perform group convolution using a split-transform-merge strategy in a simple and scalable manner. ResNext networks have shown remarkable results in applications for various computer vision tasks. The formula is as follows (Fig. 5).

$$Y=\sum _{i=1}^{C}Ti\left(x\right)$$

where $C$ denotes the cardinality, indicating the number of branches with the same topology in a module, and $Ti\left( x\right)$ represents the transformation of each branch with the same topology.

#### EfficientNet

**Fig. 6:**
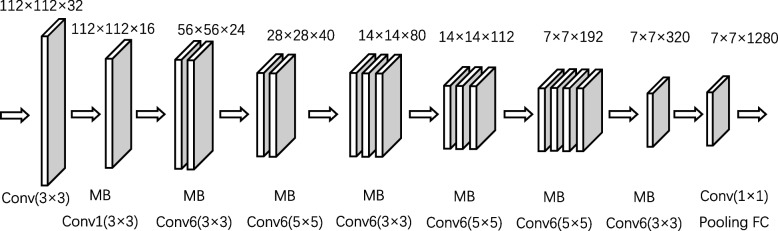
Structure of EfficientNet

EfficientNet is a new lightweight network developed by Google Research using the search technology of neural network architecture. It optimizes the three dimensions of network depth, number of channels, and resolution of input images by a fixed scale factor, which provides powerful performance of easy deployment, easy training, and high accuracy. EfficientNet is a stack of Mobile Inverted Bottleneck Convolution (MBConv), and each MBConv module contains an SE module. The SE module is a two-dimensional global pooling operation for the feature map. It transforms the high-dimensional global feature map into a low-dimensional feature vector by a compression operation to extract the channel-level global features, and then performs a nonlinear feature transformation using a multilayer perceptron (Fig. 6).

### Model training and testing

The pathology images were divided in the ratio of training set + validation set: test set = 7:3. The pathological images from the test set are diagnosed by training Resnet50, Resnext50, EfficientNet, and Densenet121 using the pathological images from the training set.

### Statistical analysis

Statistical analysis and processing were performed using SPSS 20.0 software. The receiver operating characteristic (ROC) curve was plotted accordingly to the model. The true positives (TP), false positives (FP), true negatives (TN), and false negatives (FN) were counted. And their corresponding performance metrics - recall, precision, NPV, accuracy, specificity, F1 score, Kappa coefficient, and AUC - were calculated to evaluate the diagnostic performance among the models.

## Results

**Fig. 7:**
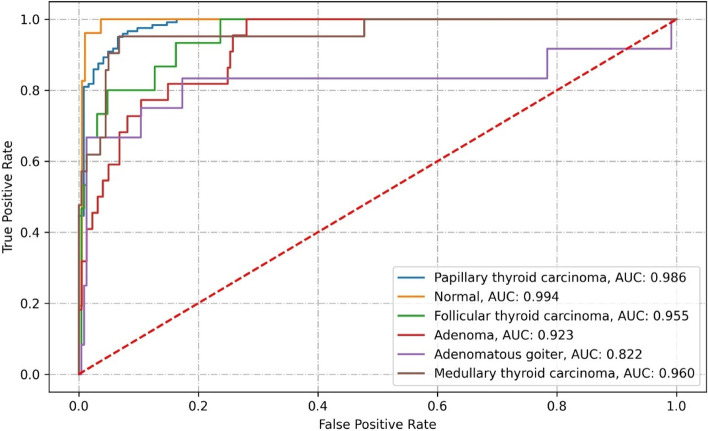
ROC curve of Resnet50

Resnet50 correctly classified 206 images from the test set. The results misclassified 37 images, which contained 2 PTC images, 5 FTC, 10 adenoma, 6 adenomatous goiter, and 14 MTC. The specific classification results and related performance indicators are detailed in Tables 1, 2, and Fig. 7.

**Table 1:** Confusion matrix of Resnet50 classification results

			Classification			
Pathological diagnosis	PTC	MTC	FTC	Adenoma	Adenomatous goiter	Normal thyroid
PTC	119(98.35%)			2(1.65%)		
MTC	9(42.86%)	7(33.33%)	2(9.52%)	3(14,29%)		
FTC	2(13.33%)		10(66.67%)	1(6.67%)	1(6.67%)	1(6.67%)
Adenoma	3(13.64%)	1(4.55%)		12(54.55%)	3(13.64%)	3(13.64%)
Adenomatous goiter			1(8.33%)	2(16.67%)	6(50.00%)	3(25.00%)
Normal thyroid						52(100.00%)

**Table 2:** Performance indicators of the Resnet50 classification

Classification	Recall	Precision	NPV	Accuracy	Specificity	F1	AUC	Kappa coefficient
PTC	98.35%	89.47%	98.18%	93.42%	88.52%	93.70%	0.981	0.7713
MTC	33.33%	87.50%	94.04%	93.83%	99.55%	48.28%	0.978	
FTC	66.67%	76.92%	97.83%	96.71%	98.68%	71.43%	0.970	
Adenoma	54.55%	60.00%	95.52%	92.59%	96.38%	57.14%	0.948	
Adenomatous goiter	50.00%	60.00%	97.42%	95.88%	98.27%	54.55%	0,889	
Normal thyroid	100.00%	88.14%	100.00%	97.12%	96.34%	93.69%	0.983

**Fig. 8:**
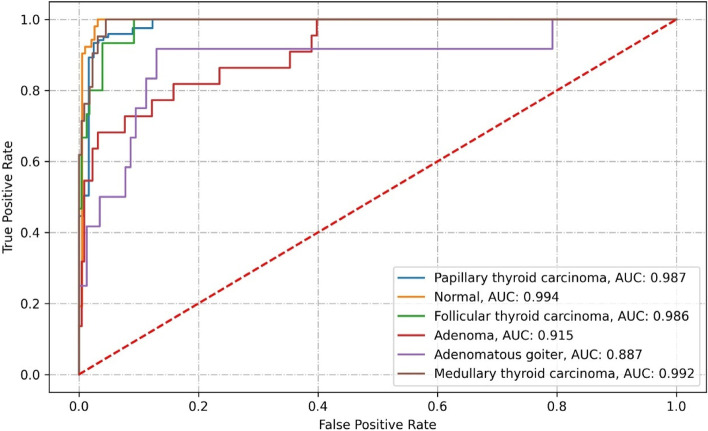
ROC curve of Densenet121

Densenet121 correctly classified 212 images from the test set. It misclassified 31 images, which contained 8 PTC images, 4 FTC, 9 adenoma, 6 adenomatous goiter, and 4 MTC. The specific classification results and related performance metrics are detailed in Tables 3, 4, and Fig. 8.

**Table 3:** Confusion matrix of Densenet121 classification results

	Classification					
Pathological diagnosis	PTC	MTC	FTC	Adenoma	Adenomatous goiter	Normal thyroid
PTC	113(93.39%)	1(0.83%)	2(1.65%)	2(1.65%)	3(2.48%)	
MTC	1(4.76%)	17(80.95%)	1(4.76%)	1(4.76%)	1(4.76%)	
FTC	1(6.67%)	1(6.67%)	11(73.33%)	1(6.67%)		1(6.67%)
Adenoma	2(9.09%)	2(9.09%)	1(4.55%)	13(59.09%)	3(13.64%)	1(4.55%)
Adenomatous goiter		1(8.33%)		1(8.33%)	6(50.00%)	4(33.33%)
Normal thyroid						52(100.00%)

**Table 4:** Performance indicators for the Densenet121 classification

Classification	Recall	Precision	NPV	Accuracy	Specificity	F1	AUC	Kappa coefficient
PTC	93.39%	96.58%	93.65%	96.72%	96.72%	94.96%	0.987	0.8146
MTC	80.95%	77.27%	98.19%	96.30%	97.75%	79.07%	0.992	
FTC	73.33%	73.33%	98.25%	96.71%	98.25%	73.33%	0.986	
Adenoma	60.00%	60.00%	95.52%	92.59%	96.38%	57.14%	0.915	
Adenomatous goiter	50.00%	46.15%	97.39%	94.65%	96.97%	48.00%	0.887	
Normal thyroid	100.00%	89.66%	100.00%	97.53%	96.86%	94.55%	0.994

**Fig. 9:**
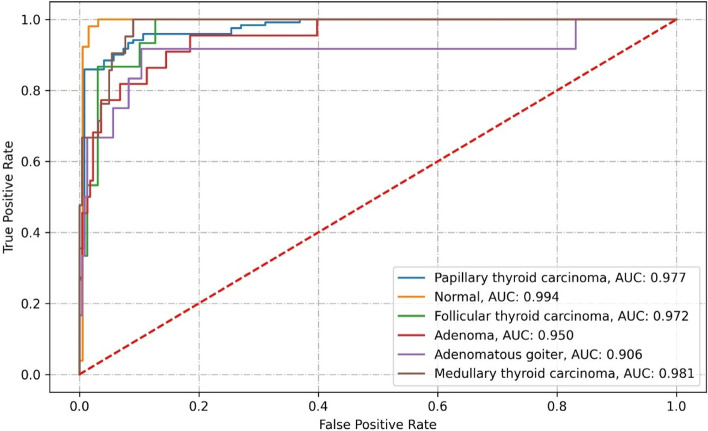
ROC curve of EfficientNet

EfficientNet correctly classified 208 images from the test set. It misclassified 35 images, which contained 8 PTC images, 1 normal thyroid, 7 FTC, 8 adenoma, 6 adenomatous goiter, and 5 MTC. The specific classification results and related performance indicators are detailed in Tables 5, 6, and Fig. 9.

**Table 5:** Confusion matrix of EfficientNet classification results

			Classification			
Pathological diagnosis	PTC	MTC	FTC	Adenoma	Adenomatous goiter	Normal thyroid
PTC	113(93.39%)	2(1.65%)	3(2.48%)	1(0.83%)	1(0.83%)	1(0.83%)
MTC	4(19.05%)	16(76.19%)		1(4.76%)		
FTC	1(6.67%)	4(26.67%)	8(53.33%)			2(13.33%)
Adenoma	6(27.27%)			14(63.64%)	2(9.09%)	
Adenomatous goiter		1(8.33%)		2(16.67%)	6(50.00%)	3(25.00%)
Normal thyroid				1(1.92%)		51(98.08%)

**Table 6:** Performance indicators of EfficientNet classification

Classification	Recall	Precision	NPV	Accuracy	Specificity	F1	AUC	Kappa coefficient
PTC	93.39%	91.13%	93.28%	92.18%	90.98%	92.24%	0.977	0.7870
MTC	76.19%	69.57%	97.73%	95.06%	96.85%	72.73%	0.981	
FTC	53.33%	72.73%	96.98%	95.88%	98.68%	61.54%	0.972	
Adenoma	63.64%	73.68%	96.43%	94.65%	97.74%	68.29%	0.950	
Adenomatous goiter	50.00%	66.67%	97.44%	96.30%	98.70%	57.14%	0.906	
Normal thyroid	98.08%	89.47%	99.46%	97.12%	96.86%	93.58%	0.994

**Fig. 10:**
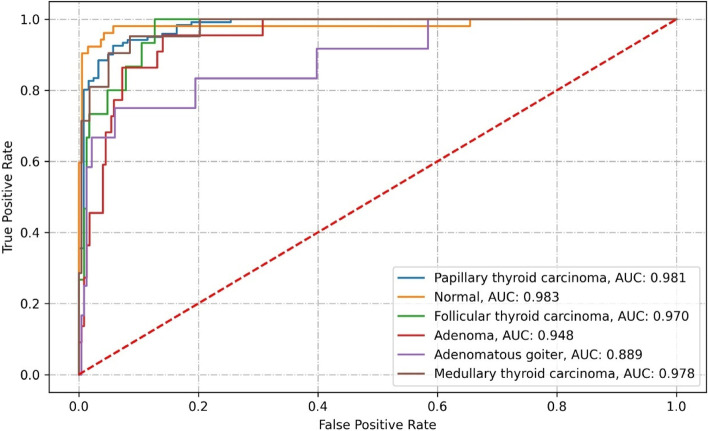
ROC curve of Resnext50

Resnext50 correctly classified 202 images from the test set. It misclassified 41, which contained 7 PTC images, 4 normal thyroid, 3 FTC, 11 adenoma, 5 adenomatous goiter, and 11 MTC. The specific classification results and related performance indicators are detailed in Tables 7, 8, and Fig. 10.

**Table 7:** Confusion matrix of Resnext50 classification results

			Classification			
Pathological diagnosis	PTC	MTC	FTC	Adenoma	Adenomatous goiter	Normal thyroid
PTC	114(94.21%)		7(5.79%)			
MTC	5(23.81%)	10(47.62%)	4(19.05%)	2(9.52%)		
FTC	2(13.33%)		12(80.00%)		1(6.67%)	
Adenoma	5(22.73%)	1(4.55%)	4(18.18%)	11(50.00%)		1(4.55%)
Adenomatous goiter				2(16.67%)	7(58.33%)	3(25.00%)
Normal thyroid				1(1.92%)	3(5.77%)	48(92.31%)

**Table 8:** Performance metrics of Resnext50 classification

Classification	Recall	Precision	NPV	Accuracy	Specificity	F1	AUC	Kappa coefficient
PTC	94.22%	90.48%	94.02%	92.18%	90.16%	92.31%	0.981	0.7508
MTC	47.62%	90.91%	95.26%	95.07%	99.55%	62.50%	0.978	
FTC	80.00%	44.44%	98.61%	92.59%	93.42%	57.14%	0.970	
Adenoma	50.00%	68.75%	95.15%	93.42%	97.74%	57.89%	0.948	
Adenomatous goiter	58.33%	63.64%	97.84%	96.30%	98.27%	60.87%	0.889	
Normal thyroid	92.31%	92.31%	97.91%	96.71%	97.91%	92.31%	0.983

## Conclusion

Resnet50, Resnext50, EfficientNet, and Densenet121 all had a high diagnostic performance. The AUC ranged from 0.822 to 0.994. The NPV, accuracy and specificity of the above four models for the diagnosis of 6 kinds of pathological images ranged from 88.52% to 100.00%, showing a stable performance. The study confirmed that the DNN model achieved satisfactory results in identifying pathological findings of thyroid tumors with a high accuracy rate. The analysis of misdiagnosed pathologies showed that the DNN model was slightly inferior to other pathological types in terms of performance in diagnosing FTC, adenoma, and adenomatous goiter. And the recall, precision and F1 score of DNN models for the diagnosis of the above three pathological images ranged from 44.44% to 80.00%. The results indicate that the DNN models has the ability to diagnose thyroid tumor pathology efficiently, but it was still insufficient in the diagnosis of FTC, adenoma and adenomatous goiter.

## Discussion

With the progress and development of science and technology, AI is becoming more and more perfect day by day. Especially in the medical field, great achievements have been made. Convolutional Neural Networks (CNN) is a class of neural networks that can perform convolutional computation with in-depth structure, and is one of the representative algorithms of DNN [5, 6]. CNN has been the core algorithm in image recognition technology and has a better performance with a large amount of data training [7]. Using this technique, images can be directly utilized in learning without the need for specialized feature extraction prior to learning, which is a breakthrough in the functionality of image recognition and classification [8]. CNNs can build a hierarchical classifier to handle a large number of image classification tasks, and CNNs can also extract features of images for other classifiers to learn for fine-grained classification [9]. In fine-grained classification, different parts of the image for feature extraction can be artificially fed into a convolutional neural network separately or can be extracted by the CNN itself through unsupervised learning [10]. Resnet50, Resnext50, EfficientNet, and Densenet121 used in this study are all CNN models, and numerous studies have confirmed their ability to efficiently identify tumor pathology images. However, CNNs are still less studied in the field of thyroid tumors, and the current studies have shown satisfactory results. For example, Wang et al. [2] also used the VGG-19 and Inception-ResNet-v2 models to classify a variety of thyroid tumor pathologies with an accuracy of 88.33% to 100%. Li et al. [11] used InceptionV3, VGG16BN, and Resnet50 to predict intraoperative frozen section pathology of thyroid nodules and correctly predicted 95.3% of benign nodules and 96.7% of malignant nodules. Other similar studies using DNN to diagnose tumor pathology have shown high accuracy rates. The accuracy of the model used in this study for diagnosing thyroid tumor pathology ranged from 92.18% to 97.53%. Similar to the results of the majority of previous studies, the final results all show that the DNN model has a high application in the classification of thyroid tumor pathological images.

The learning effect of the DNN model depends on the number and quality of images. Due to the limited number of patients eligible for enrollment, we provided more data through data augmentation, which allowed the DNN model to learn more features. In the present study, the diagnosis of FTC, adenomatous goiter, and adenoma was relatively unsatisfactory. Image analysis of misclassified pathological images and a review of the literature showed that both FTC and PTC are derived from follicular epithelial cells and have similar pathological manifestations [12], and some FTC also have papillary structures. Thus, DNN models can easily confuse them [11, 13]. Meanwhile, some MTCs have a follicular arrangement of some tumor cells, which can also lead to misdiagnosis of FTC [14]. Most of the misdiagnosed pathological images of FTC in this study were misclassified as PTC and MTC. There may be enlargement and fusion of follicles within the adenoma, forming a cystic structure [15], while PTC forms a cystic structure in some cases [16]. This results in misdiagnosis between the two. Moreover, the epithelial cell morphology of adenoma and adenomatous goiter are very similar and often appear as nodular changes under the microscope [17]. Therefore, some of the adenomas in this study were easily misdiagnosed as PTC and adenomatous goiter. Nodular-like changes are seen microscopically in adenomatous goiter. The DNN model may misclassify normal thyroid tissue when it has a large follicular structure [18, 19]. Therefore, adenomatous goiter in this study was not only easily misdiagnosed as adenoma but also partially misdiagnosed as the normal thyroid gland.

A complete pathological section includes tumor tissue, normal thyroid tissue, follicular cells, blood vessels, muscle, etc. [14]. Moreover, the differences in preparation methods and imaging equipment lead to variable representation of tissue images [20]. The pathological images used in this study were carefully selected by pathologists. The diagnosis was clear, and the DNN model performed well in diagnosing such images, but the limitation of this approach is that it limits the DNN model for atypical pathology images. We plan to include more atypical pathological pictures and collect radiomics data and metabolomics data to build models in future studies. In conclusion, this study confirms that the DNN model has high performance in the pathological diagnosis of thyroid tumors and fully demonstrates its potential in clinical applications.

### Supplementary Information

**Additional file 1.**

## Data Availability

Not applicable.
