# Supplementary material for: The value of deep neural networks in the pathological classification of thyroid tumors
Source: Diagn Pathol. 2023 Aug 19;18:95. doi: 10.1186/s13000-023-01380-2 (PMC10439627; doi:10.1186/s13000-023-01380-2)
Supplement: Supplementary file 1 — Additional file 1. [file 13000_2023_1380_MOESM1_ESM.pdf]

Retrospective Clinical Research Report

Journal of INTERNATIONAL MEDICAL RESEARCH

Journal of International Medical Research
50(4) 1–10
© The Author(s) 2022
Article reuse guidelines:
sagepub.com/journals-permissions
DOI: 10.1177/03000605221094276
journals.sagepub.com/home/imr
SAGE

Differential diagnostic value of the ResNet50, random forest, and DS ensemble models for papillary thyroid carcinoma and other thyroid nodules

Chengwen Deng[1,*], Dongyan Han[1,*], Ming Feng[2,*], Zhongwei Lv[1] and Dan Li[1]

Abstract

Objective To explore the differential diagnostic efficiency of the residual network (ResNet)50, random forest (RF), and DS ensemble models for papillary thyroid carcinoma (PTC) and other pathological types of thyroid nodules.

Methods This study retrospectively analyzed 559 patients with thyroid nodules and collected thyroid pathological images and auxiliary examination results (laboratory and ultrasound results) to construct datasets. The pathological image dataset was used to train a ResNet50 model, the text dataset was used to train a random forest (RF) model, and a DS ensemble model was constructed from the results of the two models. The differential diagnostic values of the three models for PTC and other types of thyroid nodules were then compared.

Results The DS ensemble model had the highest sensitivity, specificity, accuracy, and area under the receiver operating characteristic curve (85.87%, 97.18%, 93.77%, and 0.982, respectively).

Conclusions Compared with Resnet50 and the RF models trained only on imaging data or text information, respectively, the DS ensemble model showed better diagnostic value for PTC.

Keywords

Deep neural network, thyroid tumor, pathology, papillary carcinoma, diagnostics, artificial intelligence

Date received: 22 October 2021; accepted: 25 March 2022

[1]Shanghai Tenth People's Hospital Tongji University, Shanghai, China
[2]Tongji University, Shanghai, China

*These authors contributed equally to this work.

Corresponding author:
Dan Li, Shanghai Tenth People's Hospital Tongji University, No. 301 YanChang Road, Shanghai 200072, China.
Email: plumredlinda@163.com

Creative Commons Non Commercial CC BY-NC: This article is distributed under the terms of the Creative Commons Attribution-NonCommercial 4.0 License (https://creativecommons.org/licenses/by-nc/4.0/) which permits non-commercial use, reproduction and distribution of the work without further permission provided the original work is attributed as specified on the SAGE and Open Access pages (https://us.sagepub.com/en-us/nam/open-access-at-sage).

Introduction

The incidence of thyroid nodules is increasing annually, and the pathological types of such nodules are complex. Common malignant thyroid nodules include papillary thyroid carcinoma (PTC), medullary thyroid carcinoma (MTC), follicular thyroid carcinoma (FTC), and benign nodules including nodular goiters and adenomas. For benign thyroid nodules, only timely follow-up is required, while malignant nodules require timely surgery.[1] Among these, PTC is the most common malignant thyroid tumor, so accurately distinguishing PTC from other types of thyroid nodules is of great significance.[2] However, PTC has varying degrees of pathological similarities with MTC, FTC, adenomatous goiters, and adenomas and is easily misdiagnosed,[3] which in turn affects treatment. Therefore, improving the differential diagnosis of PTC from the other types of thyroid nodules is a clinically important research topic.

Differential diagnosis using pathological images of thyroid nodules has many challenges: (1) pathologists have different levels of professional knowledge; (2) different diagnostic results can be obtained even from the same pathological image; (3) skilled pathologists need long-term training, which conflicts with the rapidly increasing workload; and (4) overwork can result in fatigue, making pathologists more prone to misdiagnoses. The rapid development of artificial intelligence (AI) technology has overcome these problems to a certain extent. In particular, deep neural network (DNN) models have recently been shown to be efficient for pathological diagnoses[4,5] and can improve misdiagnoses caused by the lack of knowledge and fatigue of pathologists. Some studies have confirmed that the DNN models represented by Resnet50 can effectively identify the pathology of different thyroid nodules and play an increasingly prominent role in healthcare.

In recent years, the use of pathological or imaging data to train Resnet50 for diagnosis has become common, but the use of text information to train random forest (RF) models is rare. Therefore, this study used imaging data and text information to separately train residual network (Resnet)50 and RF models, and then integrated the two models to obtain a DS ensemble model. The three models were compared to explore the differential diagnostic efficiency of ResNet50, RF, and DS ensemble models for PTC and other pathological types of thyroid nodules.

Materials and methods

Patients and data

The data included in this study were obtained from patients who underwent surgical treatment or aspiration biopsy due to thyroid nodules at Shanghai Tenth People's Hospital between July 2014 and August 2021. All patients signed written informed consent. This study is a retrospective study and received exemption from the institution's review board. The reporting of this study conforms with the STARD 2015 guidelines.[6] The inclusion criteria were as follows: (1) patients who underwent initial surgical treatment or aspiration biopsy due to thyroid nodules; and (2) patients with clear pathology of thyroid nodules. The exclusion criteria were as follows: (1) patients without postoperative thyroid nodule pathology or unclear pathology; (2) patients with ^{131}I treatment; (3) patients with antitumor therapy. The pathological images and auxiliary examination results of the patients were collected to construct the pathological image and the text datasets. We have de-identified all patient details.

All pathological sections were stained with hematoxylin and eosin (HE) and observed under a DM4000B LED

microscope with intelligent automation (Leica, Wetzlar, Germany). Two senior pathologists selected the regions of interest (ROIs) and performed pathological diagnoses. The critical regions of the images where the pathology could be identified were considered the ROIs. Direct manual acquisition under a microscope was used, i.e., a Leica DFC495 microscope camera was used to directly capture pathological images. Pathological images that were controversial according to the pathologists were excluded, and all remaining pathological images were classified. The resolution ratio of images was 3264×2448 pixels, and the pixel distance of images was 264 nm/pixel (Figure 1).

PTC patients were divided into the PTC group, and patients with MTC, FTC, nodular goiters, and adenomas were grouped together in the "other" type of nodule group. The auxiliary examination results of patients included: (1) laboratory test results, such as thyroglobulin (Tg) content, thyroglobulin antibody (TgAb) content, and thyroid peroxidase antibody (TPOAb) content; and (2) ultrasound examination results, such as length of the left lymph node (mm), length of the right lymph node (mm), size of the thyroid nodule (mm), and the thyroid imaging reporting and data system (TI-RADS) classification. The reference index of laboratory tests in the hospital where the patient was treated were as follows: Tg, 3.5 to 77 ng/mL; TgAb, <100 IU/mL; and TPOAb, <40 IU/mL. Indicators were represented by 0 when within the normal range and by 1 when outside the normal range.

Data enhancement

To improve the diagnostic efficacy of the models, we performed data enhancement on the pathological image dataset. In the dataset, random flipping (horizontal flips with 50% probability), random rotation (−10° to 10°), random scaling (100%–110%), and random brightness enhancement (0%–20%) were performed to increase the amount of training data. For each image, only one of the four transformations was randomly applied.

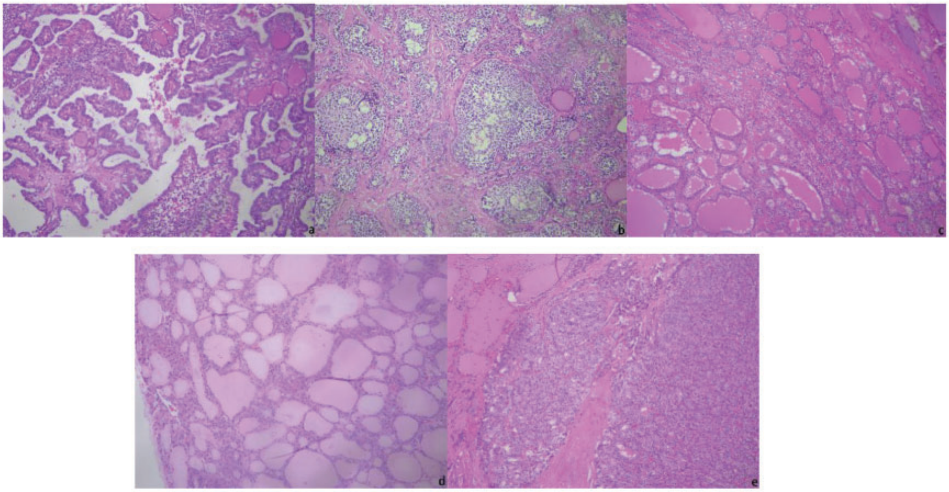

Figure 1. Pathological images of thyroid nodules: (a) Papillary thyroid carcinoma (PTC); (b) medullary thyroid carcinoma (MTC); (c) nodular goiter; (d) adenoma; (e) follicular thyroid carcinoma (FTC).

Network architecture

ResNet50 model. ResNet50 models have achieved breakthroughs in image classification. As the number of convolutional layers increases, the learning depth increases, and the effect of the model also increases. The ResNet network is modified from the visual geometry group (VGG)19 network and is constructed by adding residual blocks through the short-circuit mechanism. The main function of the residual block is to establish a short-circuit loop between the input terminal and the output terminal; therefore, when training the network, it is necessary to learn only the residuals in the previous step, rather than those in the entire process, which not only saves time from the input terminal to the output terminal but also reduces the learning difficulty of the neural network.

RF model. RF models are highly flexible machine learning algorithms that were first proposed by Leo Breiman in 2001. This method uses bootstrap sampling with replacement to repeatedly and randomly select n samples from the training sample set N to generate new training samples to train a decision tree that then generates m decision trees to form an RF. The final classification is determined by the score formed by the number of votes in the classification tree. Different from traditional decision tree algorithms, multiple decision trees are merged in a RF model. Due to the existence of multiple decision trees, a sample is finally classified into the most likely category after heavy screening and judgment, so compared with a single decision tree, RF models have the characteristics of stable performance, the ability to process a large amount of data, and accurate classifications (Figure 2).

The specific steps for an RF model are as follows: (1) bootstrap sampling with replacement is used to randomly select n samples from the sample set; (2) m features are randomly selected among all features and are used to build a decision tree; (3) a total of m decision trees are formed by repeating the above steps m times, thus forming a RF; (4) data are input, and judgment and voting for each decision tree are performed; and (5) the score of the voting ultimately determines the category of the data. The formula for RF models is:

$$H(x) = \arg\max \sum_{i=1}^{N} I(h_i(x) = Y)$$

where $h_i(x)$ denotes the decision tree classification result for each subsample i, $H(x)$ represents the classification result, Y represents the predicted variable, and $I(\cdot)$ represents the indicator function.

DS ensemble model. Supposing that m_1 and m_2 are two basic trust distribution functions, where $m_1 = \{m_1^1, m_1^2\}$ and $m_2 = \{m_2^1, m_2^2\}$; according to DS evidence theory,[7] m can be obtained by fusing m_1 and m_2. The calculation formula is:

$$m = \{m^1, m^2\},\ m^1 = \tfrac{1}{1-c} m_1^1 m_2^1,$$
$$m^2 = \tfrac{1}{1-c} m_1^2 m_2^2,\ c = \textstyle\sum_{i \neq j} m_1^i m_2^j$$

where m_1 and m_2 represent the basic trust distribution functions of Resnet50 and RF, respectively; m_1^1 and m_2^1 represent PTC; and m_1^2 and m_2^2 represent other pathological types of thyroid nodules.

Model training and testing

The RF, ResNet50, and DS ensemble models were all trained using 5-fold cross validation. For each fold of training, 60% of the data was used for training, 20% for validation, and 20% for testing. During the training process, the model with the best results in the validation set was taken and tested on the test set. The ResNet50 model was trained using the pathological imaging

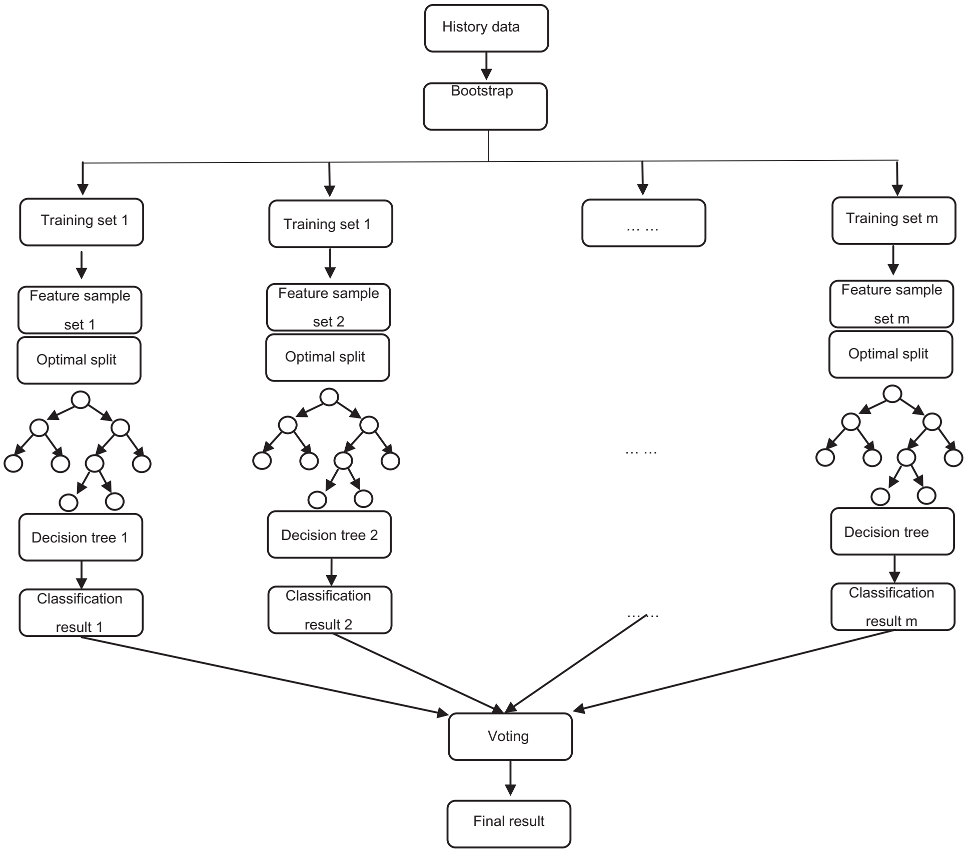

Figure 2. Random forest (RF) structure.

dataset and used to diagnose pathological images in the test set. The RF model was trained using the text dataset and used to analyze the auxiliary examination results in the test set. The results of the two models were then integrated to obtain the DS ensemble model, which was then used to diagnose PTC and other types of nodules.

Statistical analysis

SPSS 20.0 software (IBM Corp., Armonk, NY, USA) was used for all statistical analyses. Diagnoses of PTC and other types of nodules by the ResNet50, RF, and DS ensemble models were statistically analyzed. The receiver operating characteristic (ROC) curve was plotted, and the area under the ROC curve (AUC) was calculated. The diagnostic performances of different DNN models were analyzed using ROC values, with diagnostic performance represented by the AUC.

Results

This study enrolled 559 patients, including 381 with PTC, 38 with MTC, 41 with FTC, 40 with nodular goiters, and 59 with adenomas. A total of 610 pathological images were collected, including 426 of PTC, 40 of MTC, 41 of FTC, 44 of nodular goiters, and 59 of adenomas.

The ResNet50 model correctly diagnosed 546 images and misdiagnosed 64,

i.e., 35 cases of PTC were misdiagnosed as other types of nodules, and 16 cases of FTC, one case of MTC, one case of nodular goiter, and 11 cases of adenoma were misdiagnosed as PTC. The diagnostic accuracy was 89.51%, the sensitivity was 84.24%, the misdiagnosis rate was 10.49%, and the specificity was 91.78%. The diagnostic results are detailed in Table 1. ROC curve analysis showed an AUC of 0.955 (Figure 3).

Table 1. Diagnosis results of the ResNet50 model.

	Classification		
Pathology	PTC	Other types of nodules	Total
PTC	391	35	426
Other types of nodules	29	155	184
Total	420	190	610

Using auxiliary examination results, the RF model correctly diagnosed 529 cases and misdiagnosed 81, i.e., 16 cases of PTC were misdiagnosed as other types of nodules, and 18 case of FTC, 26 cases of MTC, one case of nodular goiter, and 26 cases of adenoma were misdiagnosed as PTC. The diagnostic accuracy was 86.72%, the sensitivity was 64.67%, the misdiagnosis rate was 13.28%, and the specificity was 96.24%. These diagnostic results are detailed in Table 2. ROC curve analysis showed an AUC of 0.904 (Figure 3).

The DS ensemble model correctly diagnosed 572 patients and misdiagnosed 38 cases, i.e., 12 cases of PTC were misdiagnosed as other types of nodules, and 13 case of FTC, four cases of MTC, and nine cases of adenoma were misdiagnosed as PTC. The diagnostic accuracy was 93.77%, the sensitivity was 85.87%, the misdiagnosis rate was 6.23%, and the

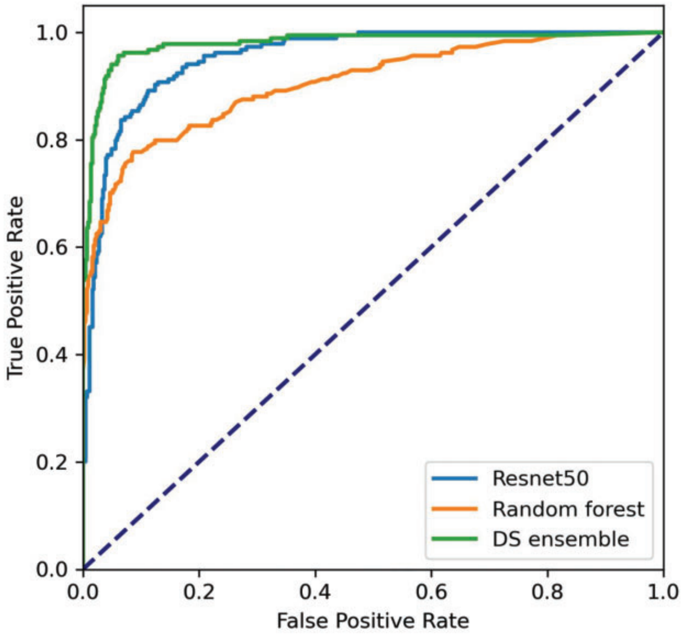

Figure 3. Receiver operating characteristic (ROC) curves of different deep neural network (DNN) models for the diagnosis of thyroid nodules.

Table 2. Diagnosis results of the RF model.

	Classification		
Pathology	PTC	Other types of nodules	Total
PTC	410	16	426
Other types of nodules	65	119	184
Total	475	135	610

Table 3. Diagnosis results of the DS ensemble model.

	Classification		
Pathology	PTC	Other types of nodules	Total
PTC	414	12	426
Other types of nodules	26	158	184
Total	440	170	610

Table 4. Comparison of the diagnosis results of DNN models.

	ResNet50	RF	DS ensemble
Sensitivity	84.24%	64.67%	85.87%
Specificity	91.78%	96.24%	97.18%
Accuracy	89.57%	86.72%	93.77%
AUC	0.955	0.904	0.979
Misdiagnose	10.49%,	13.28%	6.23%

specificity was 97.18%. The diagnostic results are detailed in Table 3. ROC curve analysis showed an AUC of 0.979 (Figure 3).

Discussion

With the progress and development of science and technology, AI is becoming increasingly skilled, especially in the medical field, where great achievements have been made. In a retrospective study, Song et al.[8] used a DNN model to predict benign and malignant thyroid nodules using ultrasound images. Using pathological results as the gold standard, the sensitivity of the DNN model was 95.2% and the specificity was 61.8%. Wang et al.[9] studied 11,715 pathological images of 806 patients with thyroid nodules, and the accuracies of identifying normal tissues, anaplastic thyroid carcinoma (ATC), FTC, MTC, PTC, nodular goiter, and adenoma after convolutional neural network (CNN) learning were 88.33%, 98.57%, 98.89%, 100%, 97.77%, 100%, and 92.44%, respectively. Recent studies have suggested that using imaging or pathological data to train DNN models can effectively identify the pathology of thyroid nodules, but there are few studies using text information to train RF models. This study used both imaging data and text information to train models, and then formed an ensemble model from the results of multiple models to explore the differential diagnostic efficacy of the ResNet50, RF, and DS ensemble models for PTC. However, the model used in this study is a relatively classic model, and the ensemble model can be achieved by calculating the mean value. More new models and other ensemble models can be used in future research, and it is expected that the application of new models may improve diagnostic efficiency.

The performance of DNN models largely depends on the quantity and quality of the dataset. A complete pathological section includes tumor tissue, normal thyroid tissue, follicular cells, blood vessels, and muscles. Moreover, different preparation methods and imaging equipment may result in different tissue features in images.[10] After the pathologist excluded pathological images with unclear diagnoses, the pathological images used by the DNN models in this study could be clearly diagnosed and had typical pathological manifestations. The DNN model was excellent at diagnosing these images, but the

limitation of this method is that it may affect the model's diagnosis of pathological images that are atypical. In future studies, the sample size should be increased to include pathological images that are atypical. Owing to the limited number of patients, data enhancement was used to provide more data and expand the dataset

The ResNet50 model was trained using pathological images and misdiagnosed 64 cases. An analysis of pathological images and a literature review showed the following results: 1) a nodular goiter may be confused with PTC because of the nodular changes and sometimes papillary structures that can be observed microscopically;[11] 2) adenomas often exhibit follicular enlargement and fusion, forming a cystic structure, while some PTC cases may also form a cystic structure, resulting in misdiagnosis between the two;[12,13] 3) both FTC and PTC are differentiated thyroid carcinomas (DTCs) from follicular epithelial cells and have similar pathological manifestations; additionally, there is a special type of PTC called follicular papillary thyroid carcinoma (FPTC) that exhibits manifestations similar to those of FTC, and FTC also has papillary structures, so DNN models easily misdiagnose the two;[14] and 4) some tumor cells in MTC may be arranged in papillary or follicular shapes, causing misdiagnoses of PTC by DNN models.[15]

The RF model was trained by the text dataset and misdiagnosed 81 auxiliary examination results. An analysis of the misdiagnosis results showed that the TgAb and Tg contents were the cause. Tg is a glycoprotein that is mainly secreted by the thyroid follicular epithelium, and its expression in the healthy human body is low.[16,17] However, patients with thyroid cancer develop inflammatory responses due to thyroid tissue damage, which may induce the activation of thyroid epithelial cells,[18] thereby causing them to release more Tg.[19] Therefore, Tg is highly expressed in patients with thyroid cancer.[20] TgAb is a Tg antibody, and an increase in Tg level can cause an increase in TgAb levels.[21] In this study, most PTC patients had TgAb > 10 IU/mL and Tg > 3.5 ng/mL, while the 16 PTC patients who were misdiagnosed as having other types of nodules had Tg < 3.5 ng/mL; therefore, the analysis of data from PTC patients with low Tg levels is prone to misdiagnoses. The 65 patients with other types of nodules who were misdiagnosed as PTC had TgAb > 10 IU/mL and Tg > 3.5 ng/mL. Because the sample size of the group with other types of nodules was smaller than that of the PTC group and the data of the PTC group accounted for a larger proportion, the DNN models may misdiagnose patients with TgAb > 10 IU/mL and Tg > 3.5 ng/mL with other types of nodules as PTC patients.

The DS ensemble model incorporated both the fusion of features and ensemble learning techniques.[22] Feature extraction on the basis of correlation analysis ensured the rationality of model data,[23] and ensemble learning on the basis of different training sets improved the accuracy of the model;[24] therefore, the DS ensemble model had high sensitivity, specificity, accuracy, and AUC. This study demonstrated the effect of using imaging data and text information to train the Resnet50, RF, and DS models and compared the Resnet50 and RF models with ensemble learning. In future studies, the sample size should be increased to improve the diagnostic efficiency of the model.

Ethics statement

The authors are accountable for all aspects of the work in ensuring that questions related to the accuracy or integrity of any part of the work are appropriately investigated and resolved. All patients were required to sign informed consent forms before the related procedures.

Acknowledgements

The authors wish to thank the anonymous referees and editors of this special issue for their constructive comments.

Author contributions

(I) Conception and design: Chengwen Deng, Dongyan Han, Ming Feng, Dan Li; (II) administrative support: Zhongwei Lv, Dan Li; (III) provision of study materials or patients: Chengwen Deng, Dongyan Han, Ming Feng; (IV) collection and assembly of data: Chengwen Deng, Dongyan Han, Ming Feng; (V) data analysis and interpretation: Chengwen Deng, Dongyan Han, Ming Feng; (VI) manuscript writing: All authors; (VII) final approval of the manuscript: all authors.

Declaration of conflicting interest

The authors have no conflicts of interest to declare.

Funding

None.

ORCID iDs

Chengwen Deng https://orcid.org/0000-0002-9782-6268
Zhongwei Lv https://orcid.org/0000-0003-3370-5560

References

1. Bakhshandeh M, Hashemi B, Mahdavi SR, et al. Evaluation of Thyroid Disorders During Head-and-Neck Radiotherapy by Using Functional Analysis and Ultrasonography[J]. *Int J Radiat Oncol Biol Phys* 2012; 83: 198–203.
2. Ancker OV, Krüger M, Wehland M, et al. Multikinase Inhibitor Treatment in Thyroid Cancer[J]. *Int J Mol Sci* 2020; 21: 10.
3. Prete A, Borges De Souza P, Censi S, et al. Update on Fundamental Mechanisms of Thyroid Cancer[J]. *Frontiers in Endocrinology* 2020; 11: 102.
4. He L, Long LR, Antani S, et al. Histology image analysis for carcinoma detection and grading[J]. *Comput Methods Programs Biomed* 2012; 107: 538–556.
5. Barker J, Hoogi A, Depeursinge A, et al. Automated classification of brain tumor type in whole-slide digital pathology images using local representative tiles[J]. *Med Image Anal* 2016; 30: 60–71.
6. Bossuyt PM, Reitsma JB, Bruns DE, et al. STARD 2015: an updated list of essential items for reporting diagnostic accuracy studies[J]. *BMJ* 2015; 351: h5527.
7. Dempster AP. Upper and lower probabilities induced by a multivalued mapping[J]. *The Annals of Mathematical Statistics* 2008; 38: 325–339.
8. Song J, Chai YJ, Masuoka H, et al. Ultrasound image analysis using deep learning algorithm for the diagnosis of thyroid nodules[J]. *Medicine* 2019; 98: e15133.
9. Wang Y, Guan Q, Lao I, et al. Using deep convolutional neural networks for multi-classification of thyroid tumor by histopathology: a large-scale pilot study[J]. *Ann Transl Med* 2019; 7: 468.
10. Halicek M, Shahedi M, Little JV, et al. Head and Neck Cancer Detection in Digitized Whole-Slide Histology Using Convolutional Neural Networks[J]. *Sci Rep* 2019; 9: 14043.
11. Li Y, Chen P, Li Z, et al. Rule-based automatic diagnosis of thyroid nodules from intraoperative frozen sections using deep learning[J]. *Artif Intell Med* 2020; 108: 101918.
12. Dov D, Kovalsky SZ, Assaad S, et al. Weakly supervised instance learning for thyroid malignancy prediction from whole slide cytopathology images[J]. *Med Image Anal* 2021; 67: 101814.
13. Halicek M, Dormer JD, Little JV, et al. Tumor detection of the thyroid and salivary glands using hyperspectral imaging and deep learning[J]. *Biomed Opt Express* 2020; 11: 1383.
14. Dolezal JM, Trzcinska A, Liao C, et al. Deep learning prediction of BRAF-RAS gene expression signature identifies noninvasive follicular thyroid neoplasms with papillary-like nuclear features[J]. *Mod Pathol* 2021; 34: 862–874.
15. Yoon J, Lee E, Koo JS, et al. Artificial intelligence to predict the BRAFV600E mutation in patients with thyroid cancer[J]. *PloS one* 2020; 15: e242806.

16. Martins-Costa MC, Maciel R, Kasamatsu TS, et al. Clinical impact of thyroglobulin (Tg) and Tg autoantibody (TgAb) measurements in needle washouts of neck lymph node biopsies in the management of patients with papillary thyroid carcinoma[J]. *Arch Endocrinol Metab* 2017; 61: 108–114.
17. Chai H, Zhu ZJ, Chen ZQ, et al. Diagnostic value of Tg and TgAb for metastasis following ablation in patients with differentiated thyroid carcinoma coexistent with Hashimoto thyroiditis[J]. *Endocr Res* 2016; 41: 218–222.
18. Wu Y, Shi X, Tang X, et al. The Correlation Between Metabolic Disorders And Tpoab/Tgab: A Cross-Sectional Population-Based Study[J]. *Endocr Pract* 2020; 26: 869–882.
19. Netzel BC, Grebe SKG, Carranza Leon BG, et al. Thyroglobulin (Tg) Testing Revisited: Tg Assays, TgAb Assays, and Correlation of Results With Clinical Outcomes[J]. *J Clin Endocrinol Metab* 2015; 100: E1074–E1083.
20. Spencer C and Fatemi S. Thyroglobulin antibody (TgAb) methods – Strengths, pitfalls and clinical utility for monitoring TgAb-positive patients with differentiated thyroid cancer[J]. *Best Pract Res Clin Endocrinol Metab* 2013; 27: 701–712.
21. Soh S and Aw TC. Laboratory Testing in Thyroid Conditions – Pitfalls and Clinical Utility[J]. *Ann Lab Med* 2019; 39: 3–14.
22. Hosni M, Abnane I, Idri A, et al. Reviewing ensemble classification methods in breast cancer[J]. *Comput Methods Programs Biomed* 2019; 177: 89–112.
23. Sudharson S and Kokil P. An ensemble of deep neural networks for kidney ultrasound image classification[J]. *Comput Methods Programs Biomed* 2020; 197: 105709.
24. Pliakos K and Vens C. Drug-target interaction prediction with tree-ensemble learning and output space reconstruction[J]. *BMC Bioinformatics* 2020; 21: 49.
